# Community perspectives on the determinants of maternal health in rural southern Mozambique: a qualitative study

**DOI:** 10.1186/s12978-016-0217-x

**Published:** 2016-09-30

**Authors:** Tabassum Firoz, Marianne Vidler, Prestige Tatenda Makanga, Helena Boene, Rogério Chiaú, Esperança Sevene, Laura A. Magee, Peter von Dadelszen, Khátia Munguambe, the CLIP Working Group

**Affiliations:** 1 Department of Medicine, University of British Columbia, 330 E. Columbia Street, New Westminister, BC V3L 3LW Canada; 2 Department of Obstetrics and Gynaecology and the Child and Family Research Institute, University of British Columbia, 950 W 28th Ave, Vancouver, British Columbia V5Z 4H4 Canada; 3 Department of Geography, Simon Fraser University, Burnaby, British Columbia V5A1S6 Canada; 4 Department of Surveying and Geomatics, Midlands State University, P Bag 9055 Gweru, Zimbabwe; 5 Centro de Investigação em Saúde da Manhiça (CISM), Bairro Cambeve, Rua 12, Distrito da Manhiça, CP 1929 Manhiça, Mozambique; 6 Department of Obstetrics and Gynaecology, St George’s, University of London, Cranmer Terrace, London, SW17 0RE UK

**Keywords:** Maternal health, Pregnancy, Determinants of health, Disparities, Poverty, Equity

## Abstract

**Background:**

Mozambique has one of the highest rates of maternal mortality in sub-Saharan Africa. The main influences on maternal health encompass social, economic, political, environmental and cultural determinants of health. To effectively address maternal mortality in the post-2015 agenda, interventions need to consider the determinants of health so that their delivery is not limited to the health sector. The objective of this exploratory qualitative study was to identify key community groups’ perspectives on the perceived determinants of maternal health in rural areas of southern Mozambique.

**Methods:**

Eleven focus group discussions were conducted with women of reproductive age, pregnant women, matrons, male partners, community leaders and health workers. Participants were recruited using sampling techniques of convenience and snow balling. Focus groups had an average of nine participants each. The heads of 12 administrative posts were also interviewed to understand the local context. Data were coded and analysed thematically using NVivo software.

**Results:**

A broad range of political, economic, socio-cultural and environmental determinants of maternal health were identified by community representatives. It was perceived that the civil war has resulted in local unemployment and poverty that had a number of downstream effects including lack of funds for accessing medical care and transport, and influence on socio-cultural determinants, particularly gender relations that disadvantaged women. Socio-cultural determinants included intimate partner violence toward women, and strained relationships with in-laws and co-spouses. Social relationships were complex as there were both negative and positive impacts on maternal health. Environmental determinants included natural disasters and poor access to roads and transport exacerbated by the wet season and subsequent flooding.

**Conclusions:**

In rural southern Mozambique, community perceptions of the determinants of maternal health included political, economic, socio-cultural and environmental factors. These determinants were closely linked with one another and highlight the importance of including the local history, context, culture and geography in the design of maternal health programs.

**Electronic supplementary material:**

The online version of this article (doi:10.1186/s12978-016-0217-x) contains supplementary material, which is available to authorized users.

## Plain english summary

The health of mothers depends on a wide variety of social, economic, environmental and political factors. We conducted a study in southern Mozambique to understand the views of women and their communities on the influence of these factors on the wellbeing on mothers. We interviewed chiefs of local administrative posts and held focus groups with women, male partners, female elders (matrons), community leaders and health workers. Based on the perspectives of each of these groups, we found that the broad range of social, economic, environmental and political factors that impact the health of mothers are influenced by each other and also by local history, context and geography. These factors should be incorporated in the design and delivery of programs for pregnant women in southern Mozambique.

## Background

The determinants of health are the conditions in which people are born, grow, live, work and age; these are shaped by the distribution of money, power and resources at global, national, and local levels [1]. These crucial influences on maternal health encompass political, economic, social, cultural and environmental dimensions [1]. Millennium Development Goal 5, while receiving unprecedented attention from governments, policy-makers, donors, researchers, civil society and other stakeholders, has not sufficiently recognised the impact of these determinants and has instead focused almost entirely on interventions delivered by the health sector [2]. The updated Global Strategy, in the context of the of the new Sustainable Development Goal (SDG) agenda, thus urges for a more integrated and transformative approach to maternal health, with much greater cross-sectoral links across social, economic and environmental pillars [1]. By widening the scope to include the determinants of health, progress on maternal health can be accelerated.

Mozambique has one of the highest rates of maternal mortality in sub-Saharan Africa with a maternal mortality ratio (MMR) of 480 per 100,000 live births [3]. Mozambique’s recent Health Sector Strategic Plan (2014–2019) comprises seven objectives based on the principles of primary health care, equity and improved quality of services [4]. Within these objectives, there is a strong focus on the determinants of health, particularly geographic inequities, nutrition and food security, access to safe water and sanitation, gender inequality, illiteracy and poverty, [4] and the importance of cross-sectoral cooperation [4]. The strategic plan is aligned with the African Union Multi-Sector Framework on Reproductive, Maternal, Neonatal and Child Health (RMNCH) that was developed to ensure integration of continental, sub-regional and country-level policy and budget action across all health and social determinant sectors [5].

The literature has identified a number of determinants that influence maternal health in Mozambique. A recent study from Maputo Province found that the high number of maternal deaths and severe maternal morbidities were influenced by lack of money for transportation and medical costs, lack of decision making power and distance from health facilities [6]. Studies from rural Mozambique confirm similar findings including spatial disparities in geographic access to reproductive health services and gender inequality in decision making in pregnancy [7, 8]. Chapman in her ethnographic work on perceived reproductive risk in central Mozambique found vulnerability was intensified by poverty, economic austerity, land shortages, increasing social conflict and inequality and lack of male support [9, 10].

However, most of the literature on the determinants of maternal health does not include the perspectives of women and their communities. Their perspectives can offer important insights into uncovering and understanding the determinants, as well as the interactions between them, and can guide the development and implementation of health interventions. Often programmes and interventions are designed without the input of those directly affected by their implementation and thus, uptake may be poor. An example from the maternal health literature of this oversight is demonstrated in a qualitative meta-synthesis examining antenatal care utilisation in low- and middle-income countries (LMIC); this study found misalignment between current antenatal care provision and the social and cultural context. [11] Given that antenatal care provision may be theoretically and contextually at odds with local beliefs and experiences, even high-quality antenatal care may not be utilized unless their views and concerns are addressed and incorporated into care [11].

One of the priority interventions from Mozambique’s National Strategic Plan for Maternal and Perinatal Mortality Reduction is to empower communities to participate actively in the process of identification and analysis of their own health problems [12]. This is supported by *Ending Preventable Maternal Mortality*, a human rights approach to maternal and newborn health, which calls for including women, girls, families and communities by enabling participation and thereby influencing how the health system works [13]. Therefore, we conducted an exploratory qualitative study in rural southern Mozambique to uncover and describe community perspectives of the determinants of maternal health and the resulting health behaviours.

## Methods

**Fig. 1:**
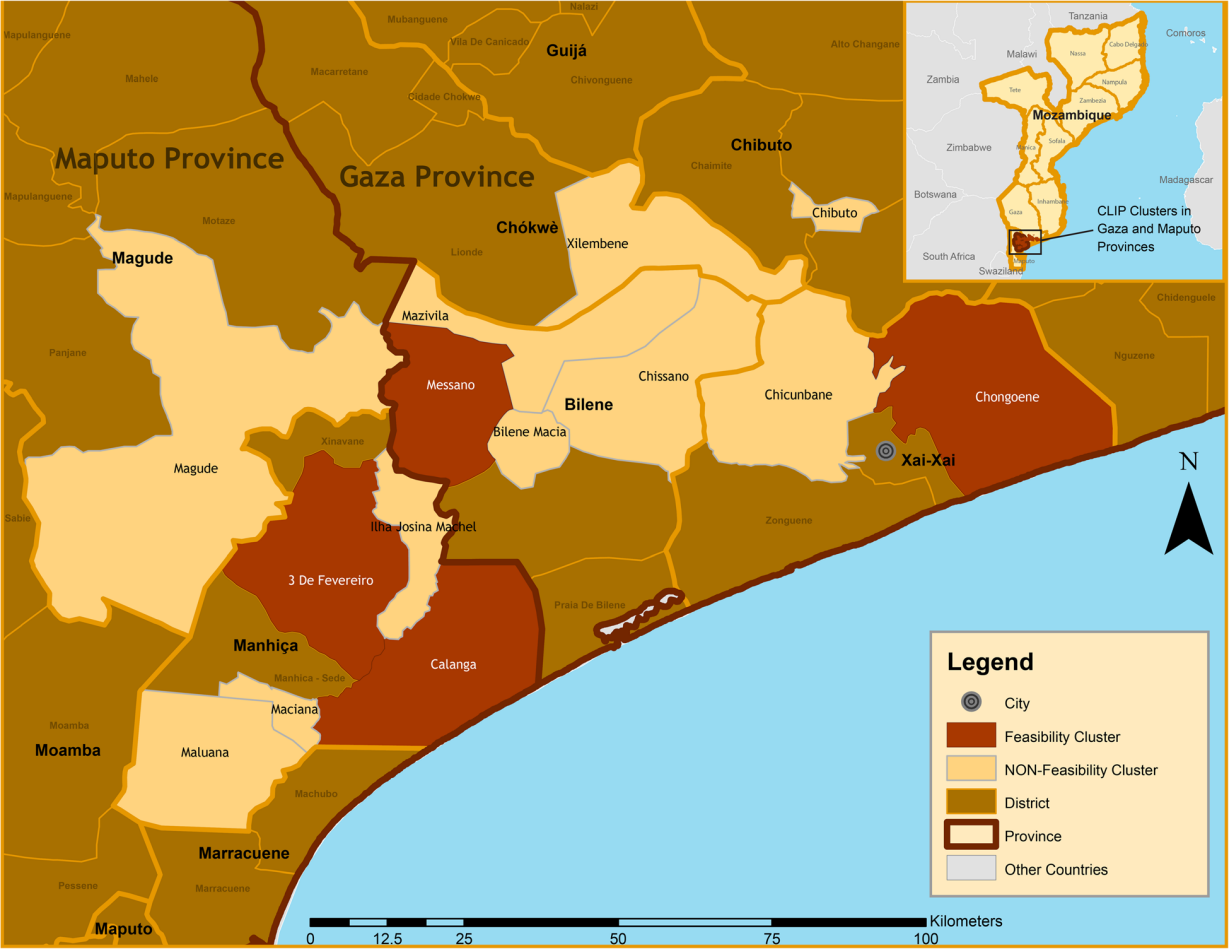
Study areas

This study was conducted as part of a large-scale mixed methods feasibility study, Community Level Intervention for Pre-eclampsia (CLIP), that aims to reduce maternal mortality and morbidity due to pre-eclampsia [14]. Within this study framework, we sought to explore the relationship between the determinants of health and maternal morbidity and mortality. To understand the context within which women live, in-depth interviews (IDI) were conducted with 12 administrative post chiefs in Gaza and Maputo (Fig. 1). The heads of administrative posts were chosen as they are familiar with the geography, politics, history and infrastructure of their communities. The recruitment process consisted of visiting each administrative post and making a formal appointment with the chief to invite them to participate in the study.

A total of 10 focus group discussions (FGD) were conducted with pregnant women, reproductive age women, matrons (elderly women in the community who serve as traditional birth attendants), male partners, community leaders, and health workers (Table 1). The study area for focus groups consisted of four administrative posts, two in Gaza and two in Maputo province (Fig. 1).

**Table 1:** Focus Group Discussions

Target group	Number of FGDs conducted
Women of reproductive age and matrons	2
Pregnant women	1
Male decision makers	2
Local health care providers: community health workers	2
Community leaders	2
Traditional healers	1

The site characteristics are described elsewhere in more detail [14, 15]. Administrative posts were purposely selected to reflect the diversity of socioeconomic and demographic characteristics in southern Mozambique, such as level of urbanization, population density, distance to a trading centre, presence of referral health facilities, and physical access to them. The administrative posts selected for this study are served by one to two primary health care centres with variable numbers of maternal child health nurses and community workers, locally named *Agentes Polivalentes Elementares* (APEs). Each administrative post had access to secondary and/or tertiary care facilities as well.

A study by our group using spatio-temporal modelling found that most women in our study area either walked or used public transport to access maternal care at the primary level, while most primary facilities provided transport to higher level facilities. 13 of the 417 communities in the study area were completely isolated from maternal health services as a result of flooding at some time during the study timeline [16].

Focus group participants were identified through community leaders after describing the inclusion and exclusion criteria and seeking permission. FGDs were usually conducted out-doors at *círculos* (the centre point of the village where the community usually gathers) or at the community leaders’ houses. Participants were recruited using sample of convenience and snow-balling. Focus groups had an average of nine participants.

The minimum number of FGDs was pre-determined based on previous experiences of reaching saturation regarding similar topics [14, 15]. Both across and within-group saturation was assessed and for this study, saturation of themes was reached. All of the administrative post chiefs were interviewed to capture singularities of administrative posts historical, political, geographical and structural contexts. Data collection was conducted between December 2013 and April 2014. This process was led by two Mozambican social scientists (KM, HB) assisted by four local interviewers. All data collectors were fluent in Portuguese and the local language, Changana. IDIs with chiefs were conducted in Portuguese whereas FGDs were conducted in Changana and Portuguese. Interviews and focus groups were translated to English by qualified translators. Signed informed consent and permission to record conversations were obtained from each participant of the IDIs and FGDs. Ethical approval for this study was obtained from the Centro de Investigação em Saúde da Manhiça (CISM) Institutional Review Board (CIBS – CISM) in Mozambique and the University of British Columbia (UBC) Clinical Research Ethics Board.

**Fig. 2:**
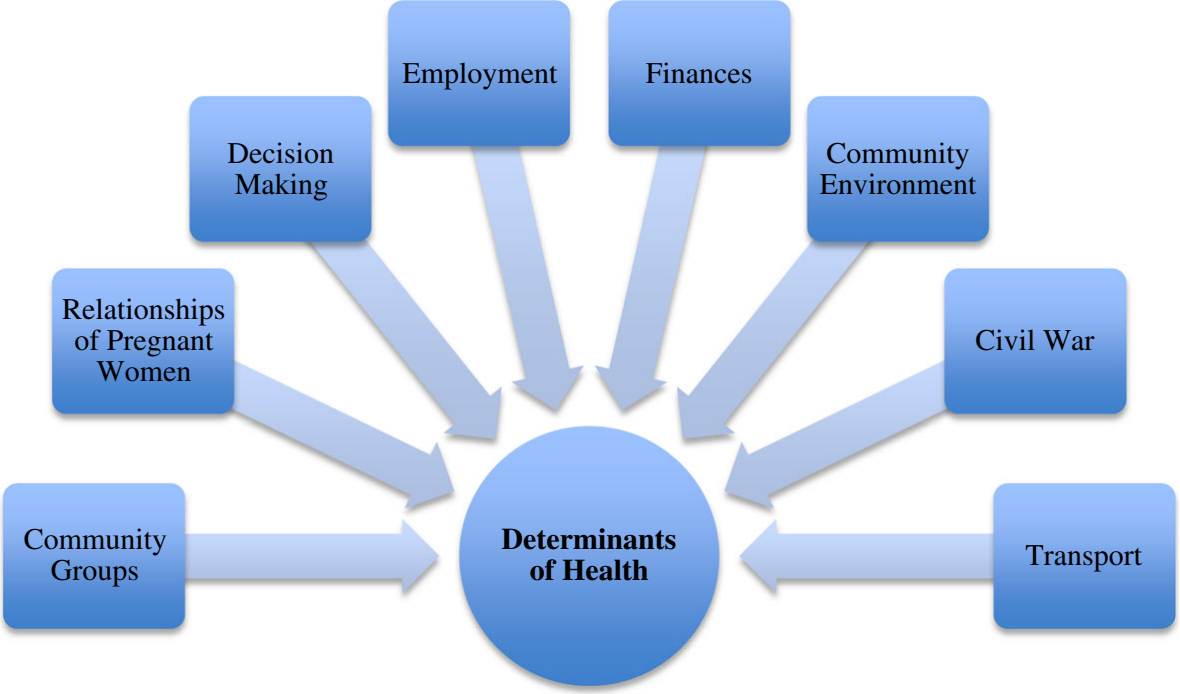
Coding structure

Three of the authors (TF, PTM and MV) from UBC coded the data. Two transcripts were coded by all three authors to confirm that there was agreement. Six transcripts were randomly selected for supplementary coding by two Mozambican social scientists (KM and HB) to ensure that the context of the text was not lost in translation. Data analysis was performed using NVivo version 10.0 (QSR International Pty. Ltd. 2012). A thematic analysis approach was taken. The thematic categories (political, economic, socio-cultural and environmental) were determined in advance based on the current literature and relevant policy frameworks. The coding structure was developed through collaboration among all researchers. New sub-themes were added as they emerged from the data. Data were analysed for the relationships between these sub-themes. Emergent propositions were tested through systematic searches of coded text and alternative explanations were explored through systematic searches of uncoded text. Fig. 2 describes the coding structure.

## Results

### Political determinants

Mozambique has a recent history of internal war and state economic and health reform policies that have had profound impact on society [6]. Thus, all administrative post chiefs in the study area were asked to describe the lasting impact of the Mozambican civil war, commonly referred to as the “Sixteen Years War”. According to participants, the war resulted in the loss of lives, infrastructure and livestock, and led to unemployment with significant impact on the practice of animal husbandry. While women, male partners, community leaders and health workers did not directly comment on the link between the war and maternal health, unemployment was consistently mentioned by women of reproductive age more than the other groups as an important factor contributing to wellbeing. Some women mentioned that they cultivated crops to make money while others mentioned that they sought domestic work (maid/housekeeper) as a means of employment. However, most women mentioned that there are no jobs.

“*Here in our community, there is no other plan, because here there is no farm to be able to wake up in the morning and go to work…. there is only hunger, that is the way we live here in our community*” [Focus group discussion with women of reproductive age, Ilha Josina, Maputo]

### Economic determinants

The lack of employment and subsequently, poverty impacted women in several ways. Women of reproductive age as well as health workers stated that financial constraints limited women’s ability to access transport and care, especially to buy medications. At the facilities, women often incurred additional expenses beyond the cost of services, such as the purchase of food while in care, however, women were frequently unable to comply due to lack of funds. Women stated that if their partners could not assume the medical expenses during pregnancy or did not share their salaries, it could lead to vulnerability or complications. Women described that at times they walked to facilities because they could not afford transport. The same observation was made by health workers, who are often frontline health care providers. Furthermore, unemployment impacted pregnant women through the inability to raise communal funds to practice *xitique*, which is a common informal savings and credit arrangement [15].

While health care workers and women had similar views about the impact of unemployment on well-being in pregnancy, male partners and community leaders were divided. Some male partners pointed out that if they were unemployed, they were unable to provide food for their pregnant wives. Other men claimed “*money does not solve anything and the one who solves is God*” [Focus group discussion with male partners, Chongoene, Gaza]. While all community leaders acknowledged that poverty was linked to difficulties in pregnancy, one mentioned that having money to purchase “tablets” was insufficient to avoid complications.

### Socio-cultural determinants

A woman’s marital status during pregnancy was identified as an important health determinant by women, health workers and community leaders. Women perceived single women (women whose partners abandoned them after finding out the pregnancy) as vulnerable particularly due to financial constraints. During focus group discussions, pregnant women described that single women have more complications because they had “*to fulfil all the requirements of the house*” [Focus group discussion with pregnant women, Manhica]. Community leaders believed widows had the greatest chance of complications because they were poor. In contrast, health workers identified divorced women as at high risk, explaining that “*it affects [them] psychologically… it even causes trouble… because she thinks, I’m pregnant but because of [lack of] sustenance, what will be of that child tomorrow, if it happens to be born”* [Focus group discussion with health workers, Chongoene, Gaza]. These respondents also felt that separated women were vulnerable because of a lack of support.

“*She does not have anybody who can support her during the gestational period, different from the woman who has a spouse. He can take her for pre-natal examination, he can take her shopping at the market and they can be together anywhere*.” [Focus group discussion with health workers, Chongoene, Gaza]

A perception shared by both women and community leaders was that relationships tended to change if women became sick during pregnancy. Reproductive age women, health workers and community leaders described that partners of sick women no longer wanted to take care of them and would at times abandon them. A community leader described a situation in his neighbourhood:

*“I, as a leader, I see a lot of things. Some people do lobola [a traditional practice of giving money, livestock, fabric etc. to the woman’s family by the husband at the time of marriage] and when she gets sick they abandon her, saying they don’t want her anymore. Even this month, we are faced with a situation of someone being hit by a motorcycle. He took her to the hospital but he no longer cares. He no longer wants to know her.”* [Focus group discussion with community leaders, Chongoene, Gaza]

At the same time, some community leaders linked pregnancy complication to a woman’s behaviour *“she gets pregnant without knowing the person who impregnated her [and] it brings complications”* [Focus group discussion with community leaders, 3 de Fevereiro, Maputo].

Women identified that for those in polygamous relationships, complications may arise if there was a bad relationship among co-spouses. They also found it stressful when male partners favoured one co-spouse over another. The relationship with in-laws was highlighted as particularly significant by several respondents. Pregnant women stated that if they were living with in-laws, they would have complications because “everyone is looking” and that “she is thinking a lot”. Health workers expressed similar opinions and stated *“there are mothers-in-law who live with the daughter-in-law while the husbands live in South Africa. A small thing, a little failure while the daughter-in-law is pregnant- this is serious trouble, and the pregnant woman can develop hypertension*”. [Focus group discussion with health workers, 3 de Fevereiro, Maputo]

Intimate partner violence was described as important factor affecting health and well-being in pregnancy.

“*Yes it happens because when the man is already angry, he no longer looks where he hits.”* [Focus group discussion with community leaders, 3 de Fevererio, Maputo]

Community leaders tended to discuss gender roles and norms in the context of intimate partner violence. They discussed that several factors would lead a man to be physically aggressive such as woman “insulting him”, “making noise” and “misusing money”. One community leader described his relationship as “*me and my wife, we have been married for forty seven years and I never beat her because she follows the rules”*. [Focus group discussion with community leaders, Chongoene, Gaza]. Male leaders did not cite reasons for physical violence but rather acknowledged that beating a pregnant woman *is “not good”* and could lead to complications such as abortion and premature delivery.

Pregnant women described a change in women’s acceptance of intimate partner violence in recent years. While in the past women were perceived as being silent about intimate partner violence, they are now are vocal about it. “*In past times she would stay at home even when beaten, still bearing children and even dying at home. But nowadays women no longer like to be beaten and there are men who, when they beat pregnant women, do not look where they hit and they can even hit in the belly. It can even be where the baby’s head is and the baby may be stillborn. In the past they would be beaten and did not return to their (maternal) homes but stayed in the household*”. [Focus group discussion with pregnant women, Manhica] They explained that women are vocal about violence and will bring up this issue in a public forum like community circles. This had consequences for the man as he could be “put in jail”. Male partners acknowledged that “*the problem of beating women is not good because women when pregnant [have] to be very well taken care of…and [beating] can provoke an unhealthy pregnancy. So it’s not good to beat them*.” [Focus group discussion with male partners, 3 de Fevereiro, Maputo]

Women of reproductive age, including pregnant women participating in the FGDs, described a complex relationship with neighbours and their immediate communities. A good relationship with neighbours was identified as being an important determinant of maternal health. Women, health workers and community leaders all felt that if women did not *“get along”* with neighbours or *“if there was no understanding”* between neighbours, it could result in difficulties or complications during pregnancy. Women described “*here when they don’t like you, they do bad things, so that when the time arrives for childbirth, you have complications, and they do things so that you are always arguing with people.”* [Focus group discussion with pregnant women, Manhinca]. Although this was not explored further in the focus groups, women’s understanding of the relationship with neighbours could be similar to Chapman’s work in which she characterized a domain of reported pregnancy illness episodes as personalistic harm caused by a human or spirit foe that women in her study called “illnesses provoked by bad spirits” [10]. She found that women reported that witchcraft and sorcery caused reproductive problems [10].

At the same time, all groups recognised that neighbours were a vital source of support for pregnant women. Community leaders mentioned “*it is not only responsibility of the pregnant woman to take care of herself, but all of us, we should help her*”. [Focus group discussion with community leaders, Chongoene, Gaza] Neighbours assisted in the event of pregnancy complications and labour, and accompanied women to health facilities. Administrative post chiefs highlighted that at times community members lend each other vehicles for transport. Matrons were acknowledged to be an especially important source of support for pregnant women. In the absence of government health workers in some rural communities, women relied on matrons for advice during pregnancy, assistance with births and accompanying them to health facilities. Matrons mentioned that they were also involved in mediating marital problems and reconciling couples.

Women identified that informal community groups were important as without them, women in these communities could not organise structured activities like *xitique*. A community health worker described the relationship in the following manner “*There are friends or adult women or neighbours with whom she must be open, with whom she must talk. She needs to have confidantes, it may be at home, it may be with neighbours, it may be at work, in the farm, at the market. She goes to look for somebody be it a friend with whom she feels free, the person that she is going to consider a confidante, isn’t it? She is going to tell her, that I am in this condition*”. [Focus group discussion with health workers, Chongoene, Gaza]

### Environmental determinants

Administrative chief posts described that the localities had faced several natural disasters including floods, droughts and cyclones. Several study areas were sandy and therefore, required large 4x4 vehicles for transport. Other areas were described as muddy or had potholes. Many of the administrative chief posts mentioned that could impede access to health services and worsen maternal health. An important consideration for accessing roads was seasonality, particularly the rainy season where many regions are prone to floods. Women identified mosquitos as an environmental factor and linked it to malaria. When asked about transportation, they mentioned that often do not have access and that vehicles are used “only if the person is serious”. Male partners also similarly identified that women walked long distances to reach facilities. They also identified that firewood smoke was a concern for pregnant women, while community leaders identified pollution as an important factor for health.

## Discussion

Our study has found that women and their communities in rural southern Mozambique identified a broad range of inter-related determinants that influence maternal health. All respondents highlighted the significance of poverty that was then described as having a number of downstream effects including the inability to pay for transport and medical costs, gender inequality and intimate partner violence, and lack of structured community groups like *xitique.* Single, divorced and widowed women, were described as a particularly vulnerable groups due to lack of financial and emotional support. At the same time, married women were vulnerable when their partners withheld money or food or if they had difficult relationships with co-spouses or in-laws.

In our study, we included discussions of the local community’s history that allowed us to contextualise findings, particularly those related to poverty and unemployment. While our study did not explore the broader impact of the war on disruption of social organisation, we found that women cited similar magnitude of impact due to unemployment and poverty resulting from the war. Our interpretation is similar to that of Chapman who described that Mozambique’s history of internal war had a profound impact on the societal structure and women’s reproductive vulnerability in her ethnographic work [9, 10]. She describes that violence, material scarcity, dislocation of rural populations, and continued male labor migration has resulted in the high burden of reproductive morbidity [9, 10].

The full health impacts of war on women’s health includes the harm and trauma during all phases of military activity that disrupt and destroy their shelter, food and health systems, their children’s education, their personal life, and their community’s cohesiveness [17]. Women are uniquely harmed by war-related disintegration of health, education and social services, by the breakdown of civil society and security, and by the loss of basic environmental assets, including potable water, sanitation, land, food, and fuel sources [17]. Women are harmed discriminately by the increased intimate partner violence within the military, as targets of rape and sexual exploitation fueled by armed conflict, and by the increased intimate partner violence that persists beyond war [17]. Programs and policies need to take a broad approach in addressing the lasting effects of war on women at multiple levels. It is critical to increase women’s participation in reconstruction by giving women access to rooms where decisions are made [18]. In post-conflict economies, tailoring education and vocational skills training towards long-term, sustainable employment will allow women to have economic independence [18]. In Mozambique and other post conflict countries, maternal health programs should include a focus on intimate partner violence which often persists beyond war.

Our findings show that maternal health programs should engage not only women but also male partners and the community at large. In our study, we found that the perspectives of community leaders varied significantly from women and male partners when it came to intimate partner violence. Community leaders appeared to have gender stereotypes about the role of women and had gendered expectations of women’s behaviours. This highlights the importance of engaging older male members such as community leaders and including discussion around gender norms and gender roles. Our study also highlights the importance of educating male partners and community leaders about birth preparedness. While women recognized the links between poverty and poor health, male partners and community leaders were divided in their perspective about the importance of money in avoiding pregnancy complications.

In addition to the political, economic and socio-cultural determinants, community informants identified several environmental factors that prohibit easy access to roads and transport thus, leading to difficulties in reaching health facilities. In Thadeus and Maine’s seminal work *Too Far To Walk,* a delay in accessing health services is described as one of the three major delays in maternal health [19]. Although women did not specifically mention distance to facility, some mentioned that due to economic constraints, they walked to facilities. A recent study from Tanzania found that large distances to hospital contribute to high levels of direct obstetric mortality [20]. Women and other community members also identified pollution and smoke as other environmental determinants which are also described in literature [21]. While there is limited published literature on maternal health and environmental determinants [21–26], key global health institutions like the World Health Organization and United Nations have been drawing attention to gender sensitive responses to the effects of the environmental, particularly climate change [27, 28].

Our study confirms the need for a broader approach to maternal health programmes. Community participation will be key in achieving a multi-sectoral approach to maternal health. The African Union advocates for the involvement of communities in the identification of maternal health problems, as well as in the planning, financing and implementation of solutions [29]. The rationale for community participation, broadly defined as members of a community getting involved in planning, designing, implementing, and/or adapting health strategies, has included responding better to communities’ needs, designing programmes that account for contextual influences on health (such as the effects of local knowledge or cultural practices), increasing public accountability for health, and it being a desirable end in itself [30].

Participatory approaches for improving maternal health have been investigated in the context of effectiveness of specific interventions, either on their own or in combined packages and most show benefits [30]. There is a paucity of literature on community participation and its effect on the determinants of health. Studies from other areas of health such as alcohol related violence, public safety and breast cancer, have shown that community participation can improve understanding of the socio-environmental causes of ill health [30]. We can draw from lessons from these other areas of health and apply them to maternal health.

## Conclusion

The political, socio-cultural, economic and environmental determinants of health are critical influences on maternal health. In rural southern Mozambique, the history of civil war has resulted in unemployment, which was recognised by community members as an important determinant of health in the community. They also identified key relationships that influence well-being in pregnancy including that with partners, co-spouses, in-laws and neighbours. Inability to access roads and transport due to the terrain, seasonality and natural disasters was highlighted as a potential environmental barrier to improved maternal health.

Frameworks for improving maternal health should include a wide array of health determinants in order to develop comprehensive strategies to reduce mortality and morbidity. Determinants not only influence access and coverage of health interventions but also shape behaviours. Programmes should address gender violence and gender inequality. It is critical to involve the community at all levels to design solutions that are appropriately targeted and contextualised. Cross-cutting multi-sectoral programme delivery is needed to effectively address and advance maternal health.

## Additional file

Additional file 1: Reviewer reports. (PDF 327 kb)

## Abbreviations

CISM: Centro de Investigação em Saúde da Manhiça
CLIP: Community level intervention for pre-eclampsia
FGD: Focus group discussions
IDI: In-depth interviews
LMIC: Low and middle-income countries
MMR: Maternal mortality ratio
RMNCH: Reproductive, maternal, neonatal and child health
SDG: Sustainable development goal
UBC: University of British Columbia
