# Supplementary material for: Community perspectives on the determinants of maternal health in rural southern Mozambique: a qualitative study
Source: Reprod Health. 2016 Sep 30;13(Suppl 2):112. doi: 10.1186/s12978-016-0217-x (PMC5056498; doi:10.1186/s12978-016-0217-x)
Supplement: Additional file 1: — Reviewer reports. (PDF 327 kb) [file 12978_2016_217_MOESM1_ESM.pdf]

Referee's comments to the authors– this sheet WILL be seen by the author(s) and published with the article

Title	Community perspectives on the determinants of maternal health in rural southern Mozambique: a qualitative study
Author(s)	Tabassum Firoz, Marianne Vidler, Prestige Tatenda Makanga, Helena Boene, Rogério Chiaú, Esperança Sevene, Laura A. Magee, Peter von Dadelszen, Khátia Munguambe
Referee's name	Bellington Vwalika

When assessing the work, please consider the following points, where applicable:

1. Is the question posed by the authors new and well defined?
2. Are the methods appropriate and well described, and are sufficient details provided to replicate the work?
3. Are the data sound and well controlled?
4. Does the manuscript adhere to the relevant standards for reporting and data deposition?
5. Are the discussion and conclusions well balanced and adequately supported by the data?
6. Do the title and abstract accurately convey what has been found?
7. Is the writing acceptable?

Please make your report as constructive and detailed as possible in your comments so that authors have the opportunity to overcome any serious deficiencies that you find and please also divide your comments into the following categories:

- Major Compulsory Revisions (which the author must respond to before a decision on publication can be reached)
- Minor Essential Revisions (such as missing labels on figures, or the wrong use of a term, which the author can be trusted to correct)
- Discretionary Revisions (which are recommendations for improvement but which the author can choose to ignore)

Where possible please supply references to substantiate your comments.

When referring to the manuscript please provide specific page and paragraph citations where appropriate.

General comments:
The paper has clarity and methodologies have been well outlined

Major compulsory revisions:

Minor essential revisions:

Discretionary revisions:

(continue on the next sheet)

Referee's comments to the authors– this sheet WILL be seen by the author(s) and published with the article

Title	Community perspectives on the determinants of maternal health in rural southern Mozambique: a qualitative study
Author(s)	Tabassum Firoz, Marianne Vidler, Prestige Tatenda Makanga, Helena Boene, Rogério Chiaú, Esperança Sevene, Laura A. Magee, Peter von Dadelszen, Khátia Munguambe
Referee's name	Stephanie Brown

When assessing the work, please consider the following points, where applicable:

1. Is the question posed by the authors new and well defined?
2. Are the methods appropriate and well described, and are sufficient details provided to replicate the work?
3. Are the data sound and well controlled?
4. Does the manuscript adhere to the relevant standards for reporting and data deposition?
5. Are the discussion and conclusions well balanced and adequately supported by the data?
6. Do the title and abstract accurately convey what has been found?
7. Is the writing acceptable?

Please make your report as constructive and detailed as possible in your comments so that authors have the opportunity to overcome any serious deficiencies that you find and please also divide your comments into the following categories:

- Major Compulsory Revisions (which the author must respond to before a decision on publication can be reached)
- Minor Essential Revisions (such as missing labels on figures, or the wrong use of a term, which the author can be trusted to correct)
- Discretionary Revisions (which are recommendations for improvement but which the author can choose to ignore)

Where possible please supply references to substantiate your comments.

When referring to the manuscript please provide specific page and paragraph citations where appropriate.

General comments: Overall, this is a well-designed study and clearly written paper. The major strength of the study is the foregrounding of women's voices about their experiences of care as evidence to guide practice. The paper provides a clear account of community engagement strategies, including the involvement of elders and community leaders, and identifies a range of social determinants that influence maternal health outcomes.

Major compulsory revisions: The main limitations of the paper, which I would encourage the authors to address in revision, are:

1. Clarification regarding the extent to which data saturation was reached (page 7, line 194 says that sample size was determined based on prior experience of reaching saturation on similar topics, but it is not clear that the authors' view was that saturation had been reached in this study.
2. Greater clarity regarding the extent and nature of differences/similarities in perspectives expressed by elders/community leaders, male decision makers, health workers vs women of reproductive age and pregnant women, and consideration of implications for local programs.

In addition, the paper would be improved by:

1. Including more detailed information in the introduction about the nature of health services accessible to women living in the setting where this study was undertaken, e.g. distance to health services, cost, modes of transport etc.
2. Broader consideration and discussion of the implications of the effects of war, lateral violence in communities, and intergenerational trauma for health services. How should local programs be tailored to address these influences

Minor corrections and typographical errors:

Page 3, line 103, determinants, is missing 's'
Page 11, line 303, affection, should be "affecting"
Page 6, line 184, please provide a definition of "matrons"
Page 6, line 186, please provide a reference for the sentence: "The site characteristics are described elsewhere."
